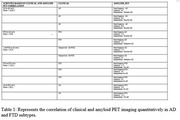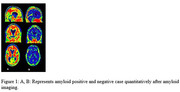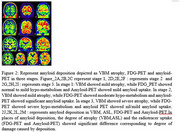# Trailing the Blaze of damage caused by Amyloid – A Multimodality, Multiparametric Amyloid MR/PET study

**DOI:** 10.1002/alz70856_098733

**Published:** 2025-12-24

**Authors:** Sandhya Mangalore

**Affiliations:** ^1^ National Institute of Mental Health and Neurosciences, Bengaluru, India

## Abstract

**Background:**

Amyloid imaging in dementia aids in detecting the deposition of amyloid plaques using different radiotracers with multimodel imaging comparision in an Indian population.

**Method:**

Twelve participants (age range: 53 – 75 years), underwent an extensive clinical assessment. In cases where FDG‐PET was available, amyloid results were co‐related. Around 350 ± 20 MBq of 18F‐NAV4694 was administered intravenously and the images were acquired using 3T Biograph mMR – a hybrid MR‐PET, 50 minutes post injection. Pre‐processing of MRI and PET were done using SPM12 and post processing of the normalised images were quantitatively analysed for standardized uptake value ratio (SUVR) and corresponding Centiloid Scale (CL) accordingly.

**Result:**

The performance of radiotracer NAV4694 is being explored for the first time in the Indian population (Asian). Standardization of in‐house radiolabeling methods for 18F NAV4694 was performed and target specific binding capacity, bio‐distribution, stability of the radiopharmaceutical were assessed and compared to look for any artifacts.

Simultaneous MR‐PET setup helps in combine data acquisition allowing depiction of structural, functional and metabolic abnormalities precisely with good spatial resolution. It gives accurate evaluation of the disease alongside many other findings with advance imaging modality such as ASL, QSM, Synthetic‐MRI, FDG‐PET, rs‐FMRI, VBM, EPSI etc. Patient were categorized as either amyloid positive or amyloid negative. Of 12 patients, the ratio of AD and FTD were 2:10 in FDG‐PET but later turned out to become 7:5 of (AD: FTD) post amyloid‐PET imaging (Figure 1,2). Our study proves that Amyloid‐PET is more sensitive than FDG‐PET. Clinically, of 12 patients, the ratio of AD and FTD which were 5:7 turned out to become 7:5 (AD:FTD) post amyloid PET scan (Table:1, Figure 1). Our study proves that Amyloid_PET is more sensitive than clinical evaluation. Co‐relation of Bloodpool, ASL, FDG‐PET and other advance imaging modalities will be discussed in detail (Figures 2,3).

**Conclusion:**

In summary, the current study demonstrated the efficacy of simultaneous MR‐PET amyloid imaging to be very similar to that of PET‐CT. NAV4694 production was standardized with GAAIN dataset and its feasibility in Indian population is established. Need for Amyloid_PET imaging as compared to FDG‐PET and Clinical evaluation is also established alongside correlation of Bloodpool and ASL imaging.